# *γ*-Aminobutyric Acid Effectively Modulate Growth Performance, Physiological Response of Largemouth Bass (*Micropterus Salmoides*) Under Combined Stress of Flow Velocity and Density

**DOI:** 10.1155/2024/9180554

**Published:** 2024-10-24

**Authors:** Yun-Jie Lin, Xu-Nan Li, Xiu-Mei Chen, Jian-Ming Chen, Xiao-Yan Jin, Jia-Xin Sun, Xiao-Tian Niu, Yi-Di Kong, Min Li, Gui-Qin Wang

**Affiliations:** ^1^College of Animal Science and Technology, Jilin Provincial Key Laboratory of Animal Nutrition and Feed Science, Key Laboratory for Animal Production, Product Quality and Safety of Ministry of Education, Jilin Agriculture University, Changchun 130118, China; ^2^Testing Center of Quality and Safety in Aquatic Product, 777 CaiYu Road, Changchun, Jilin, China; ^3^Key Laboratory of Healthy Freshwater Aquaculture, Key Laboratory of Fish Health and Nutrition of Zhejiang Province, Zhejiang Institute of Freshwater Fisheries, Ministry of Agriculture and Rural Affairs, Huzhou 313001, China

**Keywords:** *γ*-aminobutyric acid, combined stress, growth performance, largemouth bass (*Micropterus salmoides*), physiological response

## Abstract

The circular aquaculture model of largemouth bass pond engineering has the characteristics of high yield and efficiency, but it is prone to stress caused by flow velocity and density, which affects the yield of largemouth bass (*Micropterus salmoides*). *γ*-Aminobutyric acid (GABA) is believed to have the effect of improving growth and stress tolerance. We divided the largemouth bass into three groups: a control group, a flow rate and density combined stress group, and a combined stress feed supplemented with GABA (0.9%) group, and conducted a 60-day aquaculture experiment. The results showed that the final weight, weight gain rate (WGR), specific growth rate (SGR), and feed efficiency (FE) of largemouth bass significantly decreased in the combined stress group (*P* < 0.05). The serum aspartate aminotransferase (AST), alanine transaminase (ALT) activity, and glucose (GLU), malondialdehyde (MDA) level of largemouth bass significantly higher than the control group, and the serum lysozyme (LZM) activity and total antioxidant capacity (T-AOC) were significantly lower than the control group (*P* < 0.05). After adding GABA, the final weight, WGR, SGR, and FE decreased, and the serum GLU levels, AST, ALT activity, and MDA levels were downregulated, and the serum LZM activity and T-AOC of largemouth bass were upregulated. But most of the above are still at the level of the control group. Under combined stress, the messenger ribonucleic acid (mRNA) expression levels of growth hormone (GH), insulin-like growth factor-Ⅰ (IGF-I), B-cell lymphoma-2 (Blc2), nuclear transcription factor E2-related factor 2 (Nrf2), catalase (CAT), and superoxide dismutase (SOD) genes were significantly reduced (*P* < 0.05), while the mRNA expression levels of heat stress protein 70 (HSP70), heat stress protein 90 (HSP90), nuclear factor kappa-B (NF-κB), interleukin-1*β* (IL-1*β*), Bax and keap1 genes were significantly increased (*P* < 0.05). After the exogenous addition of GABA, all the above genes have a certain degree of callback, but GH, HSP70, HSP90, IL-1*β*, Bax, Nrf2, CAT, and SOD have not yet reached the level of the control group. These results indicate that adding GABA to feed can alleviate the adverse effects of combined stress of flow rate and density to a certain extent and provide insights for solving the problems in the circular aquaculture model of largemouth bass.

## 1. Introduction

Largemouth bass (*Micropterus salmoides*) is the focus of the special freshwater fish, which is native to the Mississippi River system in California, so it is also known as California bass. It has the characteristics of delicious meat quality, fast growth, rich nutrition, easy to catch, and wide temperature adaptability, known as “freshwater grouper” [1], and largemouth bass can use the whole process of artificial compound feed for breeding, has been very successfully promoted, the total production of largemouth bass breeding has reached 888,030 tons in 2023 [2]. In recent years, with the continuous expansion of the scale of aquaculture, the aquaculture models have also diversified, realizing the transformation from traditional fish breeding to modern fish breeding [3]. In particular, the pond engineering circulates aquaculture mode by increasing oxygen and accelerating water flow so that a large number of aquaculture largemouth bass like running on the “runway,” with high yield, low pollution, and other advantages [4]. This aquaculture model is commonly used in both pond and embankment bucket aquaculture, with typical flow rates ranging from 0.2 to 0.3 m/s. Studies have shown that, aside from economic benefits, the growth performance of largemouth bass is always inversely proportional to the aquaculture density, with the smallest density even being less than 0.01 kg/m^3^ [5–7]. In addition, in addition to the pollution and water purification function of this mode, circulating water is also used to “induce swimming” in farmed fish. Studies have found that “induced swimming” is beneficial for the growth of some fish, such as salmon (*Salmo salar*) and red drum (*Sciaenops ocellatus*) [8]. There are also reports that excessive flow rates can bring a series of health problems to fish [9, 10]. In fact, during the actual stocking process, due to the growth of fish, their tolerance to density and flow rate may change, which inevitably leads to stress. The survey found that largemouth bass was susceptible to flow and inventory density, causing stress and reduced performance [11]. There have been many reports demonstrating the adverse effects of flow rate or aquaculture density on fish. It reported that the feed conversion rate (FCR) rapidly increased when the velocity exceeded 35 cm/s in juvenile cobia (*Rachycentron canadum*) [12], and it was found fish spent more time on the “runway” as flow velocity increased to avoid the energy expenditure necessitated by the high-flow velocity [13]. Another Research proved that mortality was positively correlated with stocking density [14]. In the studies of juvenile flounder (*Paralichthys orbignyanus*, Valenciennes 1839), high stocking density had the capacity to stress flounder, causing higher cortisol levels [15]. Both stresses may occur simultaneously in production practice. All the above studies focus on the effects of these two stresses on fish alone, but the combined stress has not been concerned. Due to the different environmental factors, the laboratory conditions to explore the appropriate breeding density and flow speed is only a certain reference significance, so to play to the pond engineering circulating aquaculture mode of maximum production benefit, at the same time to prevent the inappropriate flow rate and density loss, using nutritional additives regulation of largemouth bass flow rate and density stress of physiological health is a worthy research direction.

The search for measures to mitigate or eliminate the negative effects caused by flow velocity or crowding stress has attracted ongoing attention. The usual measures adopted include regulating water quality conditions, scientific management and operation, reasonable breeding strategy, nutrition control means, and breeding of lines with strong stress resistance [16–19]. The addition of feed nutritional additives is one of the most direct and effective measures. Therefore, it is particularly important to develop stress-resistant feed addition through the feed route, which is also the focus of attention of many research scholars. *γ*-Aminobutyric acid (GABA), also known as 4-aminobutyric acid, is a white or near-white crystalline powder. As an important inhibitory neurotransmitter in the central nervous system, it has many functions, such as sedative nerve, anticonvulsant, regulating hormone secretion, lowering blood pressure, and improving the liver and kidney [20–23]. As a feed additive, the practice in aquatic products has proved that adding GABA in feed can improve the feed intake, resist stress, promote growth, improve feed efficiency (FE), and other aspects, achieving the purpose of reducing the occurrence of disease, reducing the risk of breeding and improving economic benefits [24–27]. GABA has emerged as a new green feed additive. Currently, most studies have shown that the addition of GABA to feed can improve FE and promote fish growth [28–30]. However, there are no reports of GABA application in largemouth bass breeding, especially the regulation of flow rate and density.

Based on the above research background, as well as combined with the preliminary test, this experiment used largemouth bass to study the effect of exogenous addition of GABA on growth performance and physiological response under the combined stress of flow velocity and density. This provides a strong basis for the healthy cultivation of largemouth bass in the circulating water system.

## 2. Materials and Methods

### 2.1. Fish

Healthy largemouth bass with consistent body size and specifications (5 ± 0.5 g) and temporarily were raised in the temperature control breeding system (oxygen static water) of Jilin Agricultural University for 15 days, feeding the basic feed and performing feed training in order to adapt to the laboratory environment. All animal experiments were approved by the Institutional Animal Care and Use Committee of Jilin Agriculture University and performed in accordance with the guidelines for experimental animals established by this committee.

### 2.2. Diets

Two diets (basic feed and test feed) were formulated with 0% and 0.9% GABA (BioXtra, ≧99%, Sigma), refer to Chen et al., [31]. The composition and nutrient levels of the basic feed and the test feed are shown in Table 1. All the dry powder raw material was passed through an 80-mesh screen and mixed evenly by formula proportion, then added the oil into the V vertical mixer mix evenly, slowly added the 20%–30% water of feed mixed again, used puffed granulation machine into 1.5 mm particle feed. After drying, all the feeds are packed and stored in −20°C refrigerator.

### 2.3. Experimental Design and Daily Management

**Figure 1:**
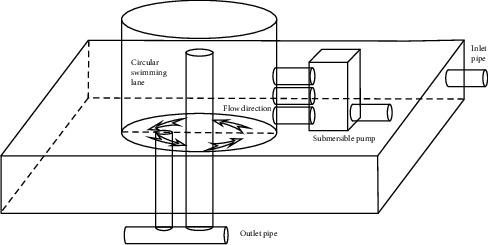
The circulating water flow rate simulation system.

After weighing, according to Table 2, largemouth bass were divided into three groups, each group three replicates, each repeated 30 largemouth bass per replicate. There were nine breeding barrels, 50 L of water storage per barrel, each equipped with a circulating water pump (Figure 1). The flow rate and the breeding density of each barrel were adjusted according to the experimental design (the flow velocity and density selected for the test were determined according to the previous laboratory study results (Appendix 1). The result is only to provide stress and does not have practical production significance due to the different practice environments). Group 1 and Group 2 fed basic feed, and Group 3 fed experimental feed.

During the experiment, feeding was conducted at 09:00 and 16:30 daily. Feed with satiety and collect leftover food to record feeding amount, and adjusted according to actual observations. Parameters such as feed intake, mortality, and water quality-related indicators were measured and recorded. The water temperature was maintained at 25−30°C, the dissolved oxygen level was above 5.0 mg/kg, the pH value was at 7.1 ± 0.1, the ammonia—N was less than 0.5 mg/L, and the nitrite content was less than 0.05 mg/L.

### 2.4. Sample Collection

After 24 h of starvation, at the end of the experiment, the number of fish was recorded, and the body weight was measured one by one. Ten largemouth basses were randomly selected for each replicate; then, the blood samples were taken from the caudal vein and anesthetized with 100 mg/L methanesulfonate-222 (MS-222). Then serum samples were collected by centrifuging and stored at −20°C refrigerator for testing. After blood collection, the liver samples of five fish from the same replicate were quickly removed and pooled on the ice, then frozen in liquid nitrogen and stored at −80°C refrigerator until analysis.

### 2.5. Growth Performance Analysis

The experimental ingredients and diets were determined by established methods based on the Association of Official Analytical Chemists (AOAC) [32]. Growth indicators were calculated as follows:

  $$\begin{array}{c} \text{Survival rate }\left(\text{SR},\%\right)=100\;\times \;\left(N_{t}/N_{0}\right), \end{array}$$

  $$\begin{array}{c} \text{Weight gain rate }\left(\text{WGR},\%\right)=100\;\times \;\left(W_{t}-\;W_{0}\right)/W_{0}, \end{array}$$

  $$\begin{array}{c} \text{Specific growth rate }\left(\text{SGR},\%/d\right)=100\;\times \;\left(\ln W_{t}-\ln W_{0}\right)/t, \end{array}$$

  $$\begin{array}{c} \text{Feed efficiency}\left(\text{FE},\%\right)=100\;\times \;\left(W_{t}-W_{0}\right)/C_{w}. \end{array}$$

  $$\begin{array}{c} \text{Feeding rate}\left(\text{FR},\%\right)=\left(100\times C_{w}\right)/\left[\left(W_{0}+W_{t}\right)/\left(2\times t\right)\right] \end{array}$$

*N*_*t*_ and *N*_0_ represent the total number of fish samples at the beginning and end of the experiment, respectively; *W*_*t*_ and *W*_0_ are the final and initial wet body mass (g) of fish, respectively; *t* is the breeding duration (day), *C*_*w*_ is the feed intake (g).

### 2.6. Serum Biochemical Parameters Analysis

The serum biochemical parameters include glucose (GLU), aspartate aminotransferase (AST), alanine transaminase (ALT), lysozyme (LZM), total antioxidant capacity (T-AOC), and malondialdehyde (MDA) were analyzed by kit following the instruction of the manufacture. All the assay kits were purchased from Nanjing Jiancheng Bioengineering Institute (Nanjing Jiancheng Bioengineering Institute, Nanjing, China).

### 2.7. The Real-Time Polymerase Chain Reaction (PCR) Analysis of Stress-Related Genes

Total RNA was extracted from the liver tissues using the TRIzol method (Takara, Dalian, China) following the manufacturer's instructions. The quantity and quality of isolated RNA were measured using a NanoDrop 2000 spectrophotometer (Thermo, NanoDrop Technologies, USA), then complementary DNA (cDNA) was synthesized with a reverse transcriptase cDNA synthesis kit (Takara, Dalian, China). The RT-PCR assays were performed on an ABI StepOnePlus Detection System (ABI 7500, USA). Using a 20 μL reaction system, FastStart Universal SYBR Green Master (ROX) (2x) 10 μL, upstream and downstream primers (10 μ mol/L) 0.6 μL each, cDNA templates for each tissue 2 μL, dd H_2_O 6.8 μL. The optimized fluorescence quantitative PCR amplification conditions were predenaturation at 95°C for 10 min, denaturation at 95°C for 15 s, annealing at 60°C for 30 s, and extension at 60°C for 1 min, with 40 cycles. Three replicates were conducted for each sample in the experiment, and dd H_2_O was used as the template for the negative control. The primer pairs used in this experiment are presented in Table 3 [33–36]. *β*-Actin was selected as a reference, and there was no difference in its expression among the different treatments. Finally, gene expression levels were calculated according to Yan et al. [37].

### 2.8. Statistical Analysis

All data were recorded in Excel and analyzed by one-way variance analysis (ANOVA) (SPSS20.0) to assess significant differences at the 5% (set *P*  < 0.05). According to the analysis results, all results were presented as means ± standard deviation (means ± SD) [38].

## 3. Results

### 3.1. Growth Performance

According to Table 4, the final weight, weight gain rate (WGR), specific growth rate (SGR), and FE of largemouth bass were significantly lower under combined stress than those in the control group (*P* < 0.05). After the exogenous addition of GABA, the final weight, WGR, SGR, and FE of largemouth bass rebounded, in which the final weight, WGR, and SGR were significantly recovered (*P* < 0.05), but still significantly lower than the control group (*P* < 0.05). However, the FE of the combined stress + GABA added group was not significantly different from the combined stress group, still significantly lower than that in the control group (*P* < 0.05). Meanwhile, there was no significant difference between the FR and survival rate (SR) in the three groups.

### 3.2. Serum Biochemical Parameters

According to Table 5. Under the combined stress, serum GLU levels, AST, ALT activity, and MDA levels were significantly increased in largemouth bass, which were significantly higher than those in the control group (*P* < 0.05). However, the serum LZM activity and T-AOC were significantly lower than that of the control group (*P* < 0.05). After the exogenous addition of GABA, serum GLU levels, AST, ALT activity, and MDA levels were downregulated in largemouth bass, while serum GLU level was still not significantly different from the combined stress, but AST, ALT activity, and MDA levels decreased significantly (*P* < 0.05), but the four indicators were still significantly higher than those of the control group (*P* < 0.05). Meanwhile, exogenous GABA addition also upregulated the serum LZM activity and T-AOC in largemouth bass, in which the LZM activity was significantly upregulated (*P* < 0.05), which was not significantly different from the control group, but the T-AOC was still significantly lower than that of the control group (*P* < 0.05).

### 3.3. Relative Messenger Ribonucleic Acid (mRNA) Expression of Stress-Related Genes

**Figure 2:**
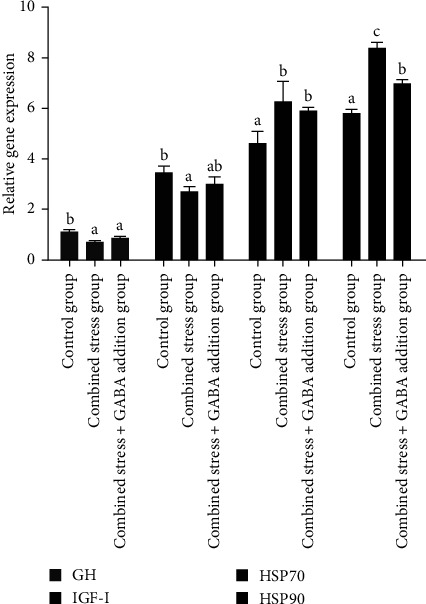
*γ*-Aminobutyric acid effectively modulates relative gene mRNA expressions of GH, IGF-Ⅰ, HSP70, and HSP90 of largemouth bass (*M. salmoides*) under combined stress of flow velocity and density (the mRNA expression of the liver of largemouth bass under fed with different treatments for 60 days. Values are means ± SD of three replicate groups, with 30 fish in each group; different letters in the same color indicate significant differences in numerical values (*r* < 0.05), as shown in the figure). GABA, *γ*-aminobutyric acid; GH, growth hormone; HSP70, heat stress protein 70; HSP90, heat stress protein 90IGF-1, insulin-like growth factor-1.

**Figure 3:**
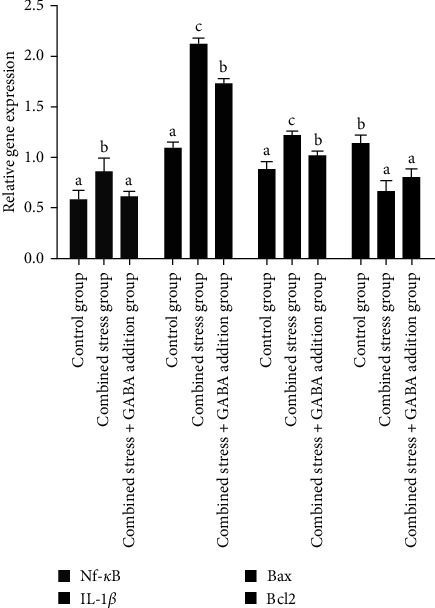
*γ*-Aminobutyric acid effectively modulates relative gene mRNA expressions of NF-*κ*B, IL-1*β*, Bax, and Bcl2 of largemouth bass (*M. salmoides*) under combined stress of flow velocity and density. Bax, bcl2-associated X; Bcl2, B-cell lymphoma-2; CAT, catalase; GABA, *γ*-aminobutyric acid; IL-1*β*, interleukin-1*β*; Nrf2, nuclear transcription factor E2-related factor 2; SOD, superoxide dismutase.

**Figure 4:**
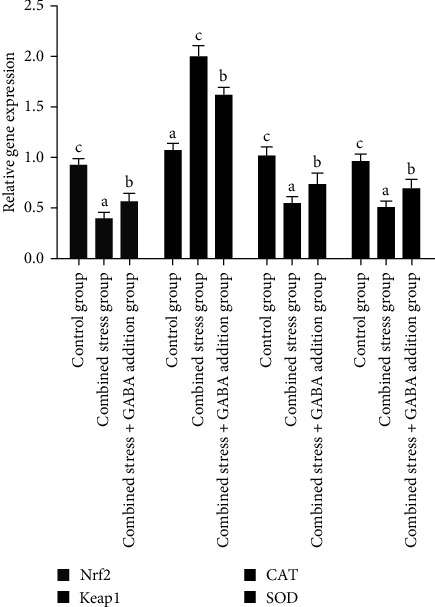
*γ*-Aminobutyric acid effectively modulates relative gene mRNA expressions of Nrf2, Keap1, CAT, and SOD of largemouth bass (*M. salmoides*) under combined stress of flow velocity and density. CAT, catalase; GABA, *γ*-aminobutyric acid; Keap1, kelch-1ike; Nrf2, nuclear transcription factor E2-related factor 2; SOD, superoxide dismutase.

According to Figure 2, the mRNA expression level of growth hormone (GH) and insulin-like growth factor-Ⅰ (IGF-I) genes decreased significantly under combined stress (*P* < 0.05), while the mRNA expression level of heat stress protein 70 (HSP70) and heat stress protein 90 (HSP90) genes increased significantly (*P* < 0.05). After the exogenous addition of GABA, the mRNA expression level of the GH and IGF-I genes increased, and the mRNA expression level of IGF-I gene was not significantly different from that of the control group, but the mRNA expression level of GH gene was still significantly lower than that of the control group (*P* < 0.05), and the mRNA expression level of HSP70 and HSP90 genes was significantly downregulated (*P* < 0.05), but still significantly higher than that of the control group (*P* < 0.05). According to Figure 3, the mRNA expression level of nuclear factor kappa-B (NF-*κ*B), interleukin-1*β* (IL-1*β*), and Bax genes increased significantly under combined stress (*P* < 0.05), the mRNA expression level of B-cell lymphoma-2 (Bcl2) gene was significantly downregulated (*P* < 0.05). After the exogenous addition of GABA, the mRNA expression level of the NF-*κ*B gene decreased significantly (*P* < 0.05), and there was no significant difference from the control group. The mRNA expression levels of IL-1*β* and Bax genes decreased significantly in the combined stress+GABA addition group (*P* < 0.05) but were still significantly higher than the control group (*P* < 0.05). The mRNA expression level of the Bcl2 gene recovered, but it was still significantly lower than that of the control group (*P* < 0.05). According to Figure 4, the mRNA expression level of nuclear transcription factor E2-related factor 2 (Nrf2), catalase (CAT), and superoxide dismutase (SOD) genes decreased significantly (*P* < 0.05), while the mRNA expression level of Keap1 gene increased significantly (*P* < 0.05). The mRNA expression levels of Nrf2, Keap1, CAT, and SOD genes were significantly adjusted (*P* < 0.05), but the mRNA expression level of Nrf2, CAT, and SOD genes was still significantly lower than that of the control group (*P* < 0.05), and the mRNA expression level of Keap1 genes was significantly higher than that of the control group (*P* < 0.05).

## 4. Discussion

### 4.1. Growth Performance

This study showed that GABA in feed supplementation significantly alleviated most physiological responses of largemouth bass under dual stress while improving its growth performance under dual stress. It is found that the inappropriate flow velocity or density can inhibit the growth of fish [31, 34]. Similar reports of unsuitable flow velocity or aquaculture density that inhibit fish growth have been reported on other fish. For example, at velocities of 0.36 m/s, juvenile turbot (*Scophthalmus maximus*) was subject to stress accompanied by a reduced growth rate [10] and the *S. salar* L. in the high-density group showed significantly lower final weight and higher ratio levels than those in medium-density and low-density groups [39]. But in practice, fish are often subject to combined stress. In this experiment, combined stress did cause the inhibition of growth in largemouth bass, which is also consistent with these above findings. It is difficult to achieve reasonable control of flow rate and density in production practice [11]. Therefore, in order to prevent the negative effects of unreasonable flow and density, the use of reasonable feed nutritional additives has become very effective. Indeed, the addition of GABA somewhat improved the growth inhibition induced by the combined stress of flow velocity and density. Reports of GABA improving the growth of aquatic animals have increased in recent years. Studies on Juvenile Pacific white shrimp (*Litopenaeus vannamei*), juvenile grass carp (*Ctenopharyngodon idellus*), and juvenile Nile tilapia (*Orechromis niloticus*) have proved that the moderate supplementation of GABA in feed can improve the growth performance [26, 40]. Previous laboratory studies on Jian carp (*Cyprinus carpiovar Jian*) have also demonstrated the addition of 90 mg/kg GABA presupplementation improved the growth [31]. Therefore, GABA addition has great potential to improve the growth performance of largemouth bass under combined stress.

### 4.2. Serum Biochemical Parameters

Improving growth is the final demonstration result, and some serum biochemical indicators also reflect the health status of the fish to some extent. Serum GLU level can be used as a stress indicator in fish [41]. Studies have shown that stressed fish sacrifice growth to promote gluconeogenesis and that GLU is an indicator of blood GLU levels, which drives a rise in plasma GLU levels [42]; GLU levels were increased in the plasma of gilthead (*Sparus auratus*), flounder (*Paralichthys olivaceus*), beef galla (*Cirrhinus mrigala*) and Senegalese sole (*Solea senegalensis*). Under high-density breeding, the effective energy may be increased under high breeding density [43–46]. Similarly, high flow rates have been reported to cause a rise in blood GLU of Japanese Sea Bass (*Lateolabrax japonicus*) and Korean rockfish (*Sebastes schlegeli*) [47]. Higher GLU levels were also detected in this study, which is perhaps the effect of crowding and flow rate stress. While detected the antioxidant capacity and immune found that the combined stress significantly increased serum AST and AST. Generally, the two enzymes have low activity, when the liver is damaged, it is released into the blood [48]. The LZM activity is associated positively with the immune function of the fish [49], and T-AOC is the sum of the antioxidant capacity of all antioxidants [50]. The combined stress also significantly downregulated serum LZM activity and T-AOC, and the oxidative damage index, MDA, increased significantly [51]. It is thus clear that combined stress caused liver damage in largemouth bass, increased oxidative stress and reduced antioxidant capacity as well as immune capacity. These results were consistent with those found on many fish species, such as Atlantic salmon [52], rainbow trout fingerlings (*Oncorhynchus mykiss*) [53], and California halibut (*Pleuronectiformes*) [54]. Despite the adverse health of largemouth bass, the addition of GABA somewhat improved this bad stress response. Specifically, it significantly reduced the levels of AST, AST, and MDA and increased the levels of LZM and T-AOC. This is also consistent with the effect of GABA addition on other aquatic animals in recent years [26, 30, 55].

### 4.3. mRNA Expression

To further analyze the improvement effect of GABA, the expression of liver genes was being explored. In this experiment, the GH and IGF-1 levels in the combined stress group were significantly lower than those in the control group, which is consistent with the growth performance of this experiment. Many studies have also shown that high stocking density reduces the expression levels of GH and IGF-Ⅰ in fish liver [56–58]. In addition, although studies have found that high-flow velocity is beneficial for fish growth, there is no doubt that too high flow rates can also increase the stress response of fish and reduce the immune capacity [9, 10]. In addition, the expression of GH and IGF-Ⅰ is not only a criterion for fish growth but also an important gene involved in fish immune function and stress. It is well known that the release of IGF-Ⅰ in the liver is regulated by GH released by the pituitary gland, which in turn is inhibited by CRH (corticotropin-releasing hormone) [59]. The decrease in GH and IGF-Ⅰ in the liver of largemouth bass in this experiment may be caused by the increase in CRH in the HPI (hypothalamus–pituitary–interrenal) stress axis under stress conditions. A research of acute ammonia stress in juvenile turbot (*S. maximus*) also confirms this point [60].

Stress can induce an oxidative stress state [61]. Under acute oxidative stress, the expression of SOD and CAT in the liver will significantly increase [62–64]. However, in this study, the expression of SOD and CAT in the combined stress group was significantly lower than that in the control group. Similar results were also observed in African catfish (*Clarias galliepinus*) and carp [65, 66], possibly due to the long-term stress state damaging the liver's antioxidant capacity. A report on chronic and acute stress also confirmed this possibility [67]. Another defense pathway of oxidative stress, consisting of Keap 1 and Nrf 2. Keap1 is an E3 ligase that induces the degradation of Nrf2 through the UPS (ubiquitin proteasome system) [68]. Under combined stress, the expression level of Nrf2 significantly decreased, while the expression level of keap1 significantly increased. The results showed that the antioxidant response of largemouth bass was severe under combined stress. Based on the data from the GABA addition group, obvious that the oxidative stress of largemouth bass was severe under combined stress.

Oxidative stress may activate different stress genes, including heat shock genes and oxidative specific genes (NF-*κ*B) [69]. The first response of the body to stimulation and pressure is to synthesize HSP to maintain homeostasis [70]. Meanwhile, heat shock activates NF-*κ*B in the liver by increasing the release of IL-1 [71]. There are binding sites for IL-1, IL-6, IL-8, etc., on NF-*κ*B, which mainly regulate immune response, inflammatory response, and cell growth in the body [72]. In this experiment, under combined stress, the expression levels of HSP70, HSP90, NF-*κ* B, and IL-1*β* in the liver of largemouth bass significantly increased as expected. At the same time, the decrease in the apoptosis gene Bcl2/Bak indicates that severe oxidative stress in the liver of largemouth bass under combined stress has induced inflammation and cell apoptosis, which is consistent with the expression of multiple studies [73, 74]. The GABA group results showed that all the genes mentioned above had a certain degree of downregulation, leading to a decrease in liver oxidative stress, inflammation, and cell damage.

Through the discussion of comprehensive growth, serum, and genes, we found that the growth and serum performance of largemouth bass decreased uniformly under the combined stress of flow rate and density, accompanied by obvious stress reactions. By mixing GABA in the feed, the growth and serum performance of largemouth bass under dual stress were improved to a certain extent, and the stress response was reduced. This study provides insights into solving the problems in the circulating water aquaculture mode of largemouth bass-intensive chemical plants.

## Figures and Tables

**Table 1:** Feed formulation and nutrient level (%, dry matter basis).

Ingredients	Group 1	Group 2	Group 3
	Basic feed		Experimental feed
Fish meal	41.00		
Corn protein powder	26.00		
Wheat bran	5.00		
Corn oil	4.00		
Wheat flour	14.00		
Dextrin	6.00		
Premix	2.00		
Calcium phosphate primary	1.50		
Choline chloride	0.50		

Total	100.00		

GABA	0.00	0.00	0.90

Proximate composition			
Crude protein (%)	41.81		41.92
Crude fat (%)	7.93		7.96
Crude ash (%)	10.77		10.57
Gross energy (MJ/kg)	17.91		18.05

*Note:* All the ingredients were obtained from Changchun Hefeng Feed Co., Ltd. Premix (mg or IU/kg diet): DL-*α*-tocopherol, 20 mg; menadione, 5 mg; thiamin, 5 mg; pyridoxine HCL, 5 mg; D-calcium pantothenate; 10 mg; nicotinic acid, 100 mg; folic acid, 2 mg; cyanocobalamin, 0.05 mg; biotin, 0.5 mg; ascorbic acid, 150 IU; p-aminobenzoic acid, 50 mg; inositol, 500 mg; choline chloride, 500 IU; retinol, 10,000 mg; cholecalciferol, 2000 mg; cobalt sulfate, 0.4 mg; copper sulfate, 5.0 mg; ferric citrate, 40 mg; magnesium oxide, 100 mg; manganous sulfate, 10 mg.

Abbreviation: GABA, *γ*-aminobutyric acid.

**Table 2:** Experimental designs.

Groups		Flow velocity (m/s)	Density (kg/m^3^)	GABA level (%)
Group 1	Control group	0	1	0
Group 2	Combined stress group	1	3	0
Group 3	Combined stress + GABA addition group	1	3	0.9

*Note:* Three replicates per group, with 30 largemouth bass per replicate.

Abbreviation: GABA, *γ*-aminobutyric acid.

**Table 3:** Real-time PCR primer sequences used in the present study.

Target genes	Forward primer	Reverse primer	*T*_*m*_ (°C)	Reference
*β*-Actin	AAAGGGAAATCGTGCGTGAC	AAGGAAGGCTGGAAGAGGG	61	[33]
GH	GAGCAGCGTCAACTCAACAA	TCAAACGATACGAGATAGACAACA	58.6	[34]
IGF-Ⅰ	CGGAGTCTCGTTCGTTATCGG	GCCTCTATCTCCACACACAAACT	58.6	[34]
HSP90	AGACAAGGGAGAAGGAAATGGAC	CGGAGCCCACATCCTCAATC	56	[35, 36]
HSP70	GTCCTACGCCTTCAACACGA	GCTGATGGTCTCGTCACACT	57	[35, 36]
NF-*κ*B	GCACGAAAACAAGAGGCATT	TCCTGAACTCGCAGAGCAAA	55	[35, 36]
IL-1*β*	AGCACCCTCGTGTCTGTTG	CAGGTTTCAACTCTGACGCT	58	[35, 36]
TNF-*α*	CTTCGTCTACAGCCAGGCATCG	TTTGGCACACCGACCTCACC	63	[33]
Caspase3	GCTTCATTCGTCTGTGTTC	CGAAAAAGTGATGTGAGGTA	54	[33]
Bax	ACTTTGGATTACCTGCGGGA	TGCCAGAAATCAGGAGCAGA	61	[33]
Bcl2	CGCCATCCACAGAGTCCT	CCGGAACAGTTCGTCTATCACC	59.4	[33]
Nrf2	CTGGTCCGAGACATACGC	CTCAGCAGACGCTCCTTC	57.5	[33]
Keap1	TATTTCCGTCAGTCCCTCAG	GGCAGCCAGCAGTTGTTC	63	[33]
CAT	GTTCCCGTCCTTCATCCACT	CAGGCTCCAGAAGTCCCACA	60.4	[33]
SOD	CCCCACAACAAGAATCATGC	TCTCAGCCTTCTCGTGGA	58	[33]

Abbreviations: Bax, bcl2-associated X; Bcl2, B-cell lymphoma-2; CAT, catalase; GH, growth hormone; HSP70, heat stress protein 70; HSP90, heat stress protein 90; IGF-Ⅰ, insulin-like growth factor-Ⅰ; IL-1*β*, interleukin-1*β*; Keap1, kelch-1ike ECH-associated protein 1; NF-*κ*B, nuclear factor kappa-B; Nrf2, nuclear transcription factor E2-related factor 2; SOD, superoxide dismutase; TNF-*α*, tumor necrosis factor-*α*.

**Table 4:** *γ*-Aminobutyric acid effectively modulates the growth performance of largemouth bass (*M. salmoides*) under combined stress of flow velocity and density.

Items	Group 1	Group 2	Group 3
	Control group	Combined stress group	Combined stress + GABA addition group
Initial weight (g)	5.08 ± 0.04^a^	5.06 ± 0.04^a^	5.04 ± 0.05^a^
Final weight (g)	35.77 ± 0.93^c^	26.74 ± 1.12^a^	30.09 ± 1.72^b^
SR (%)	100^a^	100^a^	100^a^
WGR (%)	604.47 ± 13.76^c^	428.53 ± 25.92^a^	496.78 ± 28.49^b^
SGR (%)	3.25 ± 0.03^c^	2.77 ± 0.08^a^	2.98 ± 0.08^b^
FE (%)	102.29 ± 1.56^b^	93.80 ± 1.24^a^	97.33 ± 3.68^a^
FR (%)	2.45 ± 0.05^a^	2.42 ± 0.03^a^	2.44 ± 0.06^a^

*Note:* Values followed by different lower-case letters are significantly different (*P*  < 0.05).

Abbreviations: FE, feed efficiency; FR, feeding rate; SGR, specific growth rate; SR, survival rate; WGR, weight gain rate.

**Table 5:** *γ*-Aminobutyric acid effectively modulates serum biochemical parameters of largemouth bass (*M. salmoides*) under combined stress of flow velocity and density.

Items	Group 1	Group 2	Group 3
	Control group	Combined stress group	Combined stress + GABA addition group
GLU (mmol/L)	5.0 ± 0.35^a^	5.83 ± 0.25^b^	5.68 ± 0.17^b^
AST (U/L)	5.58 ± 0.28^a^	9.79 ± 1.00^c^	7.70 ± 0.35^b^
ALT (U/L)	5.98 ± 0.30^a^	10.48 ± 1.07^c^	8.00 ± 0.24^b^
LZM (μg/L)	14.46 ± 0.92^b^	11.08 ± 0.51^a^	13.13 ± 0.94^b^
T-AOC (U/mL)	15.81 ± 1.20^b^	12.64 ± 1.22^a^	12.51 ± 1.08^a^
MDA (mmol/L)	2.95 ± 0.22^a^	4.92 ± 0.50^c^	4.03 ± 0.38^b^

*Note:* Mean values within a row with unlike superscript letters were significantly different (*P*  < 0.05).

Abbreviations: ALT, alanine transaminase; AST, aspartate aminotransferase; GLU, glucose; LZM, lysozyme; MDA, malondialdehyde; T-AOC, total antioxidant capacity.

**Table 6:** Effect of flow rate on the growth of largemouth bass.

Items	Group 0 (0 m/s)	Group 1 (0.5 m/s)	Group 2 (1.0 m/s)	Group 3 (1.5 m/s)
Initial weight (g)	5.06 ± 0.04^a^	5.07 ± 0.05^a^	5.05 ± 0.03^a^	5.05 ± 0.03^a^
Final weight (g)	17.90 ± 1.68^b^	20.57 ± 1.60^c^	16.73 ± 1.20^ab^	14.93 ± 0.85^a^
SR (%)	100^a^	100^a^	100^a^	100^a^
WGR (%)	253.66 ± 31.60^b^	305.62 ± 30.84^c^	231.23 ± 25.43^ab^	195.46 ± 15.05^a^
SGR (%)	4.20 ± 0.31^bc^	4.66 ± 0.25^c^	3.99 ± 0.26^ab^	3.61 ± 0.71^a^
FE (%)	3.39 ± 0.11^ab^	3.44 ± 0.13^b^	3.29 ± 0.08^ab^	3.22 ± 0.08^a^
FR (%)	109.70 ± 8.60^ab^	117.29 ± 8.57^b^	108.46 ± 5.85^ab^	102.30 ± 3.89^a^

*Note:* Three replicates per group, with 30 largemouth bass per replicate. Values followed by different lower-case letters are significantly different (*P*  < 0.05). The following tables are the same.

Abbreviations: FE, feed efficiency; FR, feeding rate; SGR, specific growth rate; SR, survival rate; WGR, weight gain rate.

**Table 7:** Effect of flow rate on the biochemical parameters of largemouth bass.

Items	Group 0 (0 m/s)	Group 1 (0.5 m/s)	Group 2 (1.0 m/s)	Group 3 (1.5 m/s)
GLU (mmol/L)	4.84 ± 0.34^a^	5.01 ± 0.15^a^	5.50 ± 0.25^b^	5.82 ± 0.24^b^
AST (U/L)	5.33 ± 0.10^a^	5.48 ± 0.29^a^	5.62 ± 0.28^a^	6.37 ± 0.32^b^
ALT (U/L)	5.03 ± 0.25^a^	6.06 ± 0.34a^b^	6.85 ± 0.38^b^	9.97 ± 1.02^c^
LZM (μg/L)	13.25 ± 0.86^bc^	14.51 ± 0.71^c^	11.94 ± 0.91^ab^	10.65 ± 0.47^a^
T-AOC (U/mL)	14.51 ± 1.11^b^	17.62 ± 0.86^c^	11.43 ± 0.95^a^	11.39 ± 0.68^a^
MDA (mmol/L)	1.97 ± 0.13^a^	1.67 ± 0.11^a^	3.26 ± 0.32^b^	4.00 ± 0.40^c^

Abbreviations: ALT, alanine transaminase; AST, aspartate aminotransferase; GLU, glucose; LZM, lysozyme; MDA, malondialdehyde; T-AOC, total antioxidant capacity.

**Table 8:** Effect of density on the growth of largemouth bass.

Items	Group 1 (1 kg/m^3^)	Group 2 (2 kg/m^3^)	Group 3 (3 kg/m^3^)
Initial weight (g)	5.06 ± 0.04^a^	5.07 ± 0.05^a^	5.05 ± 0.03^a^
Final weight (g)	15.77 ± 0.93^a^	18.23 ± 1.12^b^	14.70 ± 0.72^a^
SR (%)	100^a^	100^a^	100^a^
WGR (%)	211.72 ± 16.02^a^	259.58 ± 20.79^b^	190.85 ± 12.40^a^
SGR (%)	3.79 ± 0.17^a^	4.26 ± 0.19^b^	3.56 ± 0.14^a^
FE (%)	2.97 ± 0.02^a^	3.11 ± 0.11^a^	2.95 ± 0.08^a^
FR (%)	115.45 ± 1.15^b^	120.11 ± 5.38^b^	107.79 ± 0.62^a^

Abbreviations: FE, feed efficiency; FR, feeding rate; SGR, specific growth rate; SR, survival rate; WGR, weight gain rate.

**Table 9:** Effect of density on biochemical parameters of largemouth bass.

Items	Group 1 (1 kg/m^3^)	Group 2 (2 kg/m^3^)	Group 3 (3 kg/m^3^)
GLU (mmol/L)	4.60 ± 0.32^a^	5.02 ± 0.23^a^	5.64 ± 0.24^b^
AST (U/L)	5.42 ± 0.13^a^	5.69 ± 0.25^a^	6.39 ± 0.31^b^
ALT (U/L)	4.45 ± 0.22^a^	5.36 ± 0.30^b^	8.82 ± 0.90^c^
LZM (μg/L)	12.39 ± 0.81^ab^	13.56 ± 0.66^b^	11.16 ± 0.85^a^
T-AOC (U/mL)	14.51 ± 1.11^b^	17.62 ± 0.86^c^	11.43 ± 0.95^a^
MDA (mmol/L)	1.60 ± 0.10^a^	1.36 ± 0.09^a^	2.65 ± 0.27^b^

Abbreviations: ALT, alanine transaminase; AST, aspartate aminotransferase; GLU, glucose; LZM, lysozyme; MDA, malondialdehyde; T-AOC, total antioxidant capacity.

## Data Availability

Data will be made available on request.

## Appendix 1

Appendix 1 presents the preliminary experimental results for determining appropriate tassels and densities.
